# Pioglitazone Ameliorates Neuron Loss in the Cortex after Aluminum-Treatment in Rats

**DOI:** 10.1155/2015/381934

**Published:** 2015-06-08

**Authors:** Ali Rafati, Hajar Yazdani, Ali Noorafshan

**Affiliations:** ^1^Histomorphometry and Stereology Research Centre, Shiraz University of Medical Sciences, Shiraz, Iran; ^2^Physiology Department, School of Medicine, Shiraz University of Medical Sciences, Shiraz, Iran; ^3^Anatomy Department, School of Medicine, Shiraz University of Medical Sciences, Shiraz, Iran

## Abstract

The objective was evaluation of the effects of pioglitazone on medial prefrontal cortex (mPFC) of the rats exposed to aluminum (Al). Al induces structural changes in several brain regions, including mPFC. Pioglitazone is an agonist of peroxisomal proliferator activated receptor gamma. Male rats were randomly assigned to control, Al-treated (10 mg/kg/day), and Al + PIO-treated groups (Al+ 40 mg/kg/day). After 56 days, the right mPFCs were removed. Then, the volume of mPFC and its subdivisions, volume of vessels, and total number of neurons and glia were estimated using stereological methods. The results showed 13–38% decrease in the volume of the mPFC and its subdivisions, mainly in the infralimbic region (*P* < 0.02). Besides, the volume of the vessels reduced by 47% after Al-treatment (*P* < 0.02). The total number of the neurons and glial cells was also reduced (40% and 25%, resp.) in the Al-exposed rats in comparison to the control ones (*P* < 0.02). Treatment of the animals with Al + PIO ameliorated the neuron loss and no improvement was seen in other parameters (*P* < 0.02). It can be concluded that treatment of the rats with PIO could ameliorate the neuron loss in the mPFC of the Al-treated animals.

## 1. Introduction

Aluminum (Al) is an abundant metal in the environment. It is a component of cookware, utensils, medicines such as antacids, cosmetics such as deodorants, and food additives. Some foods, especially corn, yellow cheese, salt, herbs, spices, and tea, might also contain Al [1, 2]. In addition, industrialized civilizations use alum (aluminum sulfate or aluminum potassium sulfate) as flocculants in purification of drinking water. This enabled distribution of large volumes of drinking water to millions of urban consumers and allowed easy entrance of Al into the body via gastrointestinal tract [1, 2]. Al is particularly accumulated in the liver and different parts of the brain. Evidence has proved that chronic intake and metabolism of Al compounds could account for Alzheimer's disease [1, 2].

Aluminum concentrations were found to be extensive in the hippocampal region and also frontal cortex of the Alzheimer patients [3]. The hippocampal region has received a great attention. Nevertheless, a limited number of studies have assessed the effects of Al on prefrontal cortex (PFC). However, PFC is the cerebral cortex located in front of the frontal lobe. Functionally, PFC is believed to be involved in monitoring of actions, decision making, memory, motor planning, movement, and reward [4]. PFC is involved in cognition and seems to play a role in dementia associated with Alzheimer. Therefore the present study focused on the evaluation of the prefrontal cortex structure [3]. A previous study revealed reduction of glial fibrillary acidic protein levels and impairment of astrocytes function in the rats' cerebral cortex after Al-treatment [5]. Kim (2003) also showed impaired expression of neuronal nitric oxide synthase caused by exposure to Al during the early developmental stage of the brain [6].

Although different neuroprotective agents have been evaluated after Al-exposure, the present study aims to evaluate the effects of pioglitazone (PIO). PIO is an agonist of the peroxisome proliferator activated receptor. Evidence has suggested that these receptor agonists may improve some of the histopathological features of Parkinson's disease, optic nerve crush, and spinal nerve crush and cerebral ischemia [7–9]. It has been shown that activation of PPAR induces anti-inflammatory and antioxidant properties in brain. This neuroprotective influence is caused by both cerebral and vascular mechanisms. PPAR activation persuades a reduction in neuronal death by deterrence of oxidative or inflammatory processes involved in cerebral injury [10].

It has been also reported that the vascular effects are the outcome of a decrease in oxidative stress and inhibition of function of adhesion proteins, including the molecules of vascular cell or intercellular adhesion proteins injury [10]. Moreover, PPAR activation might be able to induce healing and regeneration of the vascular endothelium of the brain. In addition, there are reports of neuroprotection in chronic neurodegenerative diseases injury [10].

Expression of the PPAR-gamma has been approved in many anatomical brain regions of the adult mouse including cerebral cortex, caudate, putamen, hippocampus, thalamus, hypothalamus, and brain stem [11]. According to the above-mentioned rationales, evaluation of effectiveness of PIO in protecting the vessels, neurons, and glia in the cortex after exposure to a neurodegenerative agent (Al) can be useful for future clinical application. Since the structure of PFC after Al-exposure has received less attention, the medial PFC (mPFC) of the rats was evaluated in the first step of this study. In the second step, the protective effects of PIO on mPFC were investigated. Briefly, the study aimed to find responses to the following questions using stereological techniques: How much does the volume of the mPFC and its subdivisions change after Al-treatment? How much does the volume of the vessels change after Al-exposure? How many neurons and glial cells of the mPFC are lost after Al-consumption? Does PIO protect the mPFC structure and its subdivisions after exposure to Al? Does PIO protect the vessels, neurons, and glial cells of mPFC after exposure to Al?

## 2. Methods

### 2.1. Animals and Treatments

In this study, 15 adult male Sprague-Dawley rats (165 ± 15 g) were obtained from the Laboratory Animal Center of Shiraz University of Medical Sciences. The Ethics Committee of the University approved the animal experiment (Approval number 92-6789). The animals were housed under standard conditions, room temperature (22–24°C), and a 12:12 h light-dark schedule and had free access to water and food. The animals were divided into three groups (*n* = 5). Five animals per group were sufficient for the stereological studies and were chosen according to Hyde et al. (2007) [12].

Control group (I) received i.p. injection of 1 mL normal saline (as a vehicle) daily, (II) Al group received i.p. injection of 1 mL of the vehicle containing 10 mg/kg/day aluminum chloride (Sigma Aldrich, Germany) [13, 14], and (III) Al + PIO group received 40 mg/kg/day of pioglitazone (Sigma Aldrich, Germany) in addition to Al [15]. PIO was administrated by gavages. The treatments were continued for 56 days.

The rationale for selecting the Al dose was the aluminum intake in adults which is usually 10 mg/kg/day, but it will increase to hundreds mg/kg/day when people receive foods with a high aluminum concentration or aluminum-containing drugs. Therefore the exact intake of Al cannot be determined exactly in human being in different countries [16]. Finally, the dose of Al was selected according to European Food Safety Authority. The Authority reported that the mean dietary contact from water and food in nonoccupational exposed human adults exhibited large variations between different countries and, within a country, between different studies. The measure was reported to range from 1.6 to 13 mg Al per day [13, 14]. In addition, it should be mentioned that the selected dose has been recommended in animal models to induce neuronal degeneration. It is important in the present research to evaluate the protective effects of PIO on loss of neuronal and glial cells after exposure to the neurodegenerative dose.

The rationale for choosing 40 mg/kg dose for pioglitazone was based on the research by Almasi-Nasrabadi et al. [15]. They administrated 10, 20, and 40 mg/kg of PIO to mice receiving scopolamine and found that dose of 40 mg/kg improved some behavioral performances of the mice [15].

### 2.2. Tissue Preparation

The rats were anesthetized with ketamine-xylazine (80 and 20 mg/kg, resp.). After transcardial perfusion of the rats with buffered formaldehyde, the brains were uncovered by an incision along the midline of the skull. After that, the right cerebral hemisphere was immersed in buffered formaldehyde for one week and then embedded in the paraffin block. mPFC was recognized according to the atlas of Paxinos and Watson. It located at 4.70–2.70 mm ventral and 4.70–2.70 mm dorsal to the bregma [17]. A complete series of coronal sections of 4 *μ*m followed by 26 *μ*m thickness were obtained and continued along the whole length of the mPFC. Overall, about 8–12 sections with 4 *μ*m thickness and 8–12 sections with 26 *μ*m thickness were selected from each mPFC in a systematic random manner. The sections were stained with cresyl violet (0.1% in distilled water) in order to estimate the volume of the mPFC and its subdivisions, volume of the vessels, and total number of the neurons and glial cells. It should be mentioned that the glial cells were distinguished from the neurons by their smaller size and lack of a nucleolus and stained cytoplasm.

### 2.3. Estimation of the Volume of mPFC and Its Subdivisions

mPFC includes three subdivisions, namely, prelimbic (PL), infralimbic (IL), and Anterior Cingulate Cortex (ACC) [17]. Using a projecting microscope, the live images of 4 *μ*m thickness sections were evaluated at the final magnification of 24x according to the rat brain atlas [17]. The boundaries of the mPFC were considered from the most frontal section where the underlying white matter appeared and was sustained on every mounted section up to the presence of the genu of the corpus callosum where decussating fibers could be seen. The volumes were calculated using the Cavalieri method [18, 19]. Briefly, a grid of points was overlaid on the PFC images and the volume of the mPFC was estimated by the following formula:

(1) $$\begin{array}{c} V\left(\;\text{mPFC}\;\right)=\sum P\left(\;\frac{a}{p}\;\right)d, \end{array}$$

where “∑*P*” was the total points hitting the mPFC sections (here 99–135 points per animal), “*a*/*p*” was the area associated with each point (here was 0.1 mm^2^), and “*d*” was the distance between the sampled sections.

The volume density of the vessels and their lumens, “*V*_*v*_(vessels/mPFC),” was estimated using point-counting method and the following formula [18, 19]:

(2) $$\begin{array}{c} V_{v}\left(\;\text{vessels}/\text{mPFC}\;\right)=\frac{P\left(\text{vessels}\right)}{P\left(\text{mPFC}\right)}, \end{array}$$

where “*P*(vessels)” and “*P*(mPFC)” represented the total number of the points on the vessels profile and the mPFC, respectively. The total volume was estimated by multiplying the *V*_*v*_(vessels/mPFC) by *V*(mPFC) [18, 19].

### 2.4. Estimation of the Number of Neurons and Glia

**Figure 1:**
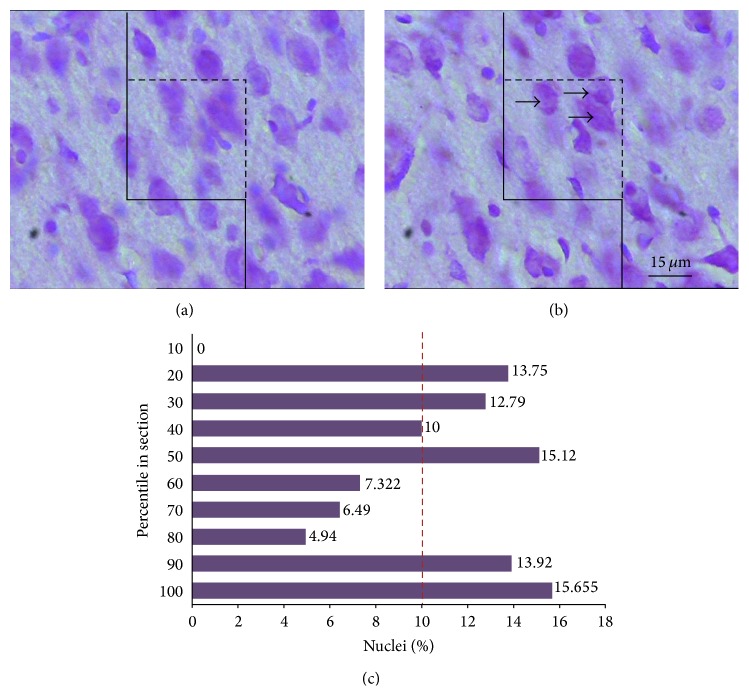
Optical disector method. (a) Look-up section of the disector. (b) Reference section. The cells whose nucleoli appeared in the reference section and did not touch the left and lower borders of the frame were counted. (c) The plot of the *z*-axis distribution of the nuclei. Each histogram indicates the percentile of the counted nuclei in ten percent of the section thickness.

The total number of the neurons and glial cells was determined using the optical disector method at the final magnification of 3400x on 26 *μ*m thickness sections [18, 19]. A 100x oil immersion objective lens was used, as well. The location of the microscopic fields was carefully chosen by systematic uniform random sampling while moving the stage in identical distances in *x*- and *y*-directions. An unbiased counting frame with inclusion and exclusion borders was overlaid on the images of the computer monitor to avoid the “edge effect” and biased counting of the cells (Figure 1). The focal plane of the microscope was moved downwards in *z*-direction. A microcator was attached to the stage of the microscope to measure the *z*-axis movement in depth of the section. The upper and lower guard zones were considered to avoid counting the cutting artifacts located at the upper and lower surfaces of the tissue sections. The height of the disector was demarcated as the section thickness excluding the 4 *μ*m thick guard zones at the top and bottom of each section. Any nucleolus (neurons) or nucleus (glial cells) coming into maximal focus within the height of the disector was selected if it lay completely or partly inside the counting frame and did not hit the exclusion lines (Figure 1). The suitable guard zone was defined after estimating the percent of nuclei in the ten columns of the *z*-axis thickness [20]. Each column represented 10 percent of the section thickness. According to the histogram, the upper and lower 20% were considered as the guard zones. Besides, the remaining columns were considered as the height of the disector (Figure 1). The numerical density (*N*_*v*_) was estimated using the following formula [18, 19]:

(3) $$\begin{array}{c} N_{v}=\frac{\sum Q}{\left[\left(\sum P\left(a/f\right)h\right)\right]}\left[\;\left(\;\frac{t}{\text{BA}}\;\right)\;\right], \end{array}$$

where “∑*Q*” was the number of the neurons or glial cells nuclei coming into focus and counted (on the average, 80–101 neurons and 100–136 glial cells were counted per mPFC), “∑*P*” was the total number of the counting frames in all fields, “*a*/*f*” was the area per frame (470 *μ*m^2^), “*h*” was the height of the disector, “*t*” was the real section thickness measured using the microcator when the *Q* was counted (here ~20 *μ*m on the average), and “BA” was the block advance of the microtome which was set at 26 *μ*m. The total number of the neurons was estimated by multiplying the numerical density (*N*_*v*_) by *V*(mPFC) [18, 19].

### 2.5. Estimation of the Coefficient of Error (CE)

The CE(*V*) for the estimate of the mPFC volume was calculated using the following formula [18, 19]:

(4) CEV=∑P−1·12403∑Pi2+∑PiPi+2−4∑PiPi+1+0.0724·ba·n∑Pi1/2,

where “*b*” and “*a*” represented the mean section boundary length and mean sectional area, respectively. The CE for the estimate of the total number of neurons, CE(*N*), was calculated using CE(*V*) and CE(*N*_*v*_) as follows:

(5) CEN=CE2Nv+CE2V1/2CENv=nn−1∑Q−2∑Q−2+∑P2∑P2−2∑Q−P∑Q−∑P1/2.

### 2.6. Statistical Analysis

The study data were entered into the SPSS statistical software (version 15.0) and analyzed using Kruskal-Wallis and Mann-Whitney *U* test with adjusted alpha level. *P* ≤ 0.05 was considered statistically significant.

## 3. Results

### 3.1. Volume of the mPFC and Its Parts

**Figure 2:**
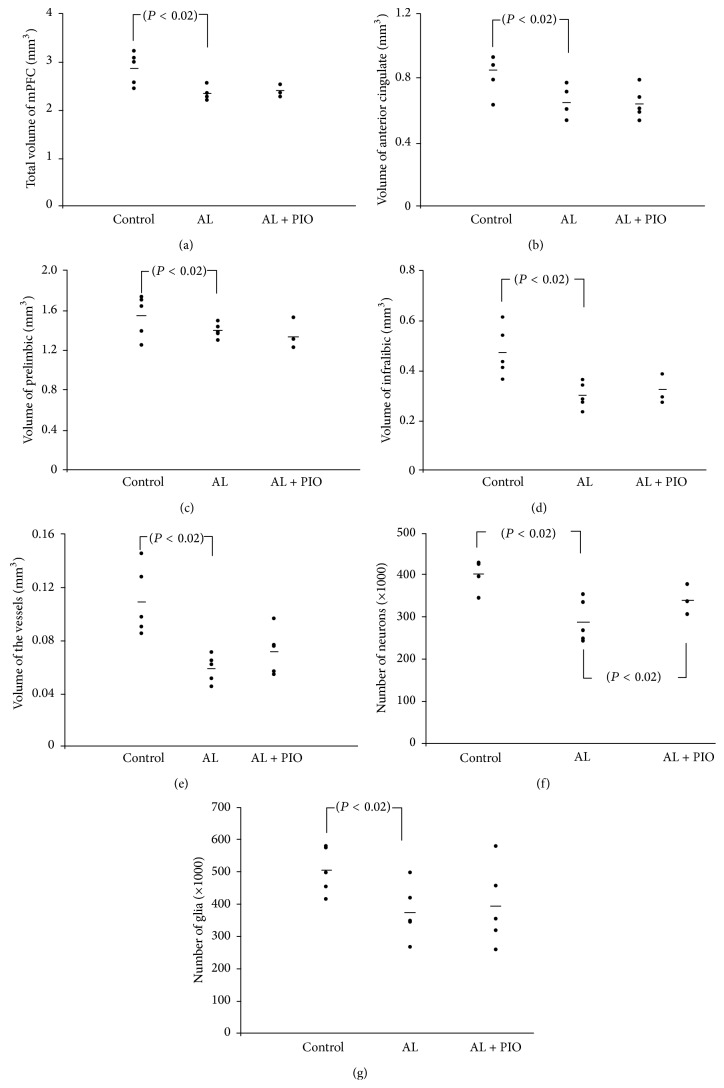
The scattered plots of the total volume of the mPFC (a), anterior cingulate cortex (b), prelimbic cortex (c), infralimbic cortex (d), vessels (e), number of neurons (f), and number of glial cells (g) in the control, Al, and Al + PIO groups.

CE was 0.03 for estimation of the volume of the mPFC that shows acceptable value. The study results revealed ~17% decrease in the volume of the mPFC in the Al group in comparison to the control rats (*P* < 0.02) (Figure 2). However, no improvement was seen in Al + PIO group in comparison to the AL ones. The volume of the ACC reduced by 22% in the Al-treated rats in comparison to the control ones (*P* < 0.02). However, no significant difference was observed between the Al + PIO and Al groups regarding the volume of the ACC (Figure 2). The study findings showed ~13% reduction in the volume of the prelimbic region in the Al group in comparison to the control animals (*P* < 0.02) (Figure 2). Nonetheless, no amelioration was detected in the PL volume in the Al + PIO group in comparison to the AL rats. The study results revealed ~38% decrease in the volume of the infralimbic region in the Al-treated rats in comparison to the control ones (*P* < 0.02) (Figure 2). Nevertheless, the volume of the infralimbic region remained unchanged in the Al + PIO-treated rats compared to the Al-treated animals. The volume of the vessels reduced by 47% in the Al-treated rats in comparison to the control ones (*P* < 0.02). However, no improvement was found in the vessels' volume in the Al + PIO group compared to the Al-treated rats (Figure 2).

### 3.2. Total Number of the Neurons and Glia

CE was 0.11 and 0.12 for estimation of the number of neurons and number of glial cells, respectively, which shows acceptable values. The results showed that the total number of the neurons in the mPFC was significantly reduced by 40% in the Al group in comparison to the control rats (*P* < 0.02). However, the number of the neurons was significantly higher in the Al + PIO group in comparison to the Al-treated animals. Although there was a difference between the control and Al groups, PIO ameliorated the effects of Al on reduction of the number of neurons. In other words, the number of the neurons reduced in the Al + PIO group, but to a lesser extent compared to Al alone (*P* < 0.02) (Figure 2). The results showed that the total number of the glial cells in the mPFC was significantly reduced by 25% in the Al group in comparison to the control rats (*P* < 0.02). Yet, further analysis revealed no improvement in the Al + PIO animals (Figure 2).

### 3.3. Qualitative Evaluation

**Figure 3:**
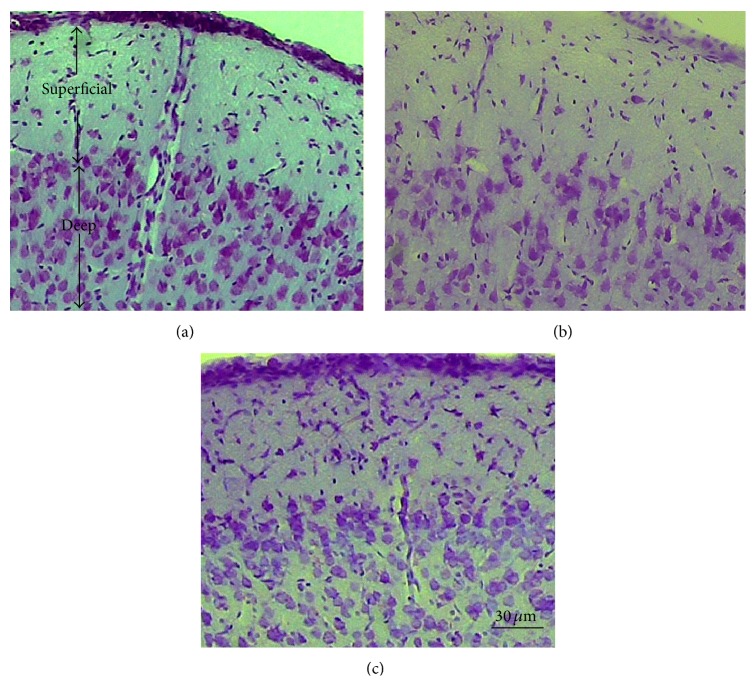
Photomicrograph of the mPFC in the rats. (a) Control, (b) Al-treated, and (c) Al + PIO-treated groups. The difference between the cells population in the superficial and deep layers of the cortex can be observed in the groups.

As Figure 3 depicts, compared to the control rats, accumulation of the neurons and glia was lesser in the Al-treated animals in both superficial and deep layers of the mPFC. More population of the cells was detected after the concomitant treatment of the rats with PIO and Al in comparison to administration of Al in the layers of the cortex.

## 4. Discussion

The present study evaluated the effects of Al on the structure of the mPFC of the rats using stereological methods. The protective effects of PIO on the Al-treated rats were investigated, as well. Reduction of the volume of mPFC and its subdivisions was the finding of the first step of this survey. The cortex volume reduction is in coincidence with the work of Stoeckel et al., (2013). Their MRI findings suggested medial frontal cortex atrophy in patients with mild Alzheimer disease [21].

Cerebral cortex including mPFC is composed of neurons, glial cells, vessels, and neuropil. The vessels volume mainly reflects the blood volume that can fill the vessels and, therefore, the blood supply of the tissue. This parameter has received less attention in Al-exposure before. Our study results showed that this parameter was decreased after Al-treatment in the rats. Our finding in regard of vessels density coincides with Chen et al. (2013) who evaluated the brain changes using MRI and estimation of cerebral blood flow [22]. They reported that regional cerebral blood flow and the density of the vessels are reduced after AlCl_3_-induced Alzheimer disease in rats [22]. Their results showed that in these animals most vessels around the hippocampus and cortex could not be observed [22]. The results obtained by Bhattacharjee et al. (2013) suggested that the endothelial cells that lined the cerebral vasculature might have biochemical properties leading to binding of Al to them [23]. There are researches that suggested that cerebral hypoxia triggers the potential downstream inflammatory and pathogenic consequences [23, 24]. Therefore, the above-mentioned studies might explain the reduction of the vessels' volume in the present study.

Another finding of this study was loss of neurons and glial cells. These findings were in agreement with those obtained in the previous studies, demonstrating that Al added to the diet was able to induce neuron and glia loss and Alzheimer's disease [25, 26]. The evidences of Walton (2009) suggested aluminum progressively collects in cortical and limbic areas of vulnerable subjects' brains, eventually producing cell loss and disrupting afferent and efferent circuitry [27].

Previous studies claimed that glial cells might be one of the targets of Al neurotoxicity [5]. Reduction of the vessels' volume and consequently the blood supply might be one of the reasons for the cell loss in the Al-exposed rats. In addition, Bhattacharjee et al. (2014) reported disruption in the blood brain barrier in the vessels of the nervous system after Al toxicity [26] that might be followed by Al distribution in the brain tissue. AL toxicity may finally lead to degradation of the cells. Apoptosis and necrosis are the main mechanism of cell death in Al toxicity [28, 29].

The results of the current study confirmed that pioglitazone diminished neurodegeneration caused by Al. Up to now, a limited number of studies have indicated the structural protection of PIO and most studies have focused on behavioral improvement by PIO. The neuroprotective results of the current study were consistent with a previous evaluation of the neuroprotective effects of PIO, in which 30 mg/kg PIO administration in a rat model of Parkinsonism protected the neurons [30]. Additionally, Pang et al. (2014) conducted an in vitro study and demonstrated that PIO could protect the rats' cerebellar granule cells against nutrient deprivation [31]. Moreover, R. Gupta and L. K. Gupta (2012) demonstrated that PIO offered protection against memory dysfunctions observed in Alzheimer's model [32]. They suggested that the protective action of PIO was possibly due to its antioxidant action. One other study also confirmed that PIO had a neuroprotective effect against scopolamine-induced cholinergic system deficit and cognitive impairment [33]. PIO is essentially used for management of diabetes mellitus type 2 either alone or in combination with other drugs [30–33]. PIO decreases blood sugar levels. Yet, neuroprotection should be added to its effects, as well. The mechanism of action of PIO might be due to its anti-inflammatory process. It has been suggested that PIO inhibits the inflammatory response by attenuating the mediators of inflammation, including expression of cyclooxygenase-2, (an enzyme responsible for inflammation, COX-2), prostaglandin E2 generation (a bioactive lipid that provokes an extensive range of biological effects associated with inflammation, PGE2), and microglia activation, resulting in protection of neurons [30–33].

## 5. Conclusion

In conclusion, Al could induce structural changes in the rats' mPFC. Besides, pioglitazone showed beneficial effects on neuronal protection in the Al-exposed animals.
